# Urban ambient air pollution and blood pressure in schoolchildren in Accra, Ghana

**DOI:** 10.1038/s41598-026-54135-6

**Published:** 2026-06-04

**Authors:** Abosede S. Alli, Kate A. Kyeremateng, James Nimo, Carissa L. Lange, Nancy Dery, Prince Anku, Lilly Y. Adzrakor, Geofrey K. Kotoka, Philomena Oppong, Samuel Agyei-Mensah, Youssef Oulhote, Allison F. Hughes, Majid Ezzati, Raphael E. Arku

**Affiliations:** 1 Department of Environmental Health Sciences, School of Public Health and Health Sciences, University of Massachusetts, Amherst, MA USA; 2 Department of Biostatistics and Epidemiology, School of Public Health and Health Sciences, University of Massachusetts, Amherst, USA; 3 Department of Physics, University of Ghana, Legon, Ghana; 4 Department of Environmental & Sustainable Engineering, State University of New York, Albany, USA; 5 Department of Geography and Resource Development, University of Ghana, Accra, Ghana; 6 Department of Environmental Medicine and Public Health, Icahn School of Medicine at Mount Sinai, New York, USA; 7 Department of Epidemiology and Biostatistics, School of Public Health, Imperial College London, London, UK; 8 MRC Centre for Environment and Health, School of Public Health, Imperial College London, London, UK; 9 Regional Institute for Population Studies, University of Ghana, Accra, Ghana; 10 Imperial Global Ghana, Accra, Ghana

**Keywords:** Air pollution, Fine particulate matter, Black carbon, Hypertension, Children, Ghana, Diseases, Environmental sciences, Health care, Risk factors

## Abstract

Ambient air pollution has been linked to elevated blood pressure (BP) in adults, but research is limited among children, particularly in sub-Saharan Africa (SSA). We investigated the potential effects of ambient fine particulate matter (PM_2.5_), black carbon (BC), and nitrogen dioxide (NO_2_) exposures on BP in school-aged children in Accra, Ghana’s capital and one of the fastest growing cities in SSA. We performed a cross-sectional analysis among 919 (60% girls) schoolchildren aged 7–14 years across 90 elementary schools. Following NIH guideline, we define elevated BP as age, sex, and height specific systolic and/or diastolic BP values at ≥ 90th percentile. PM_2.5_, BC, and NO_2_ concentrations at homes and schools were estimated using spatiotemporal land-use regression models developed specifically for Accra using measurement data from 146 sites. Covariate-adjusted associations were estimated using multivariable mixed-effects linear and logistic regression models. The mean SBP and DBP among the children were 107.2 (9.7) and 69.1 (7.1) mmHg, respectively, with one-third of the children having elevated BP. Children’s PM_2.5_ exposure at both home and school were 5–8 times the World Health Organization (WHO) annual guideline of 5 µg/m^3^, BC levels were above the maximum value in the “good practice statement for BC” (5.1 µg/m^3^), and NO_2_ concentrations exceeded the WHO annual guideline of 10 µg/m^3^ by 11-fold at some locations. Overall, PM_2.5_, BC, and NO_2_ were negatively associated with BP after adjustment for demographic and lifestyle covariates. For instance, a 10 unit increase in air pollution at homes was associated with a small decrease in SBP (PM_2.5_: −0.70 mmHg (95% CI −3.34, 1.94), BC: −4.61 mmHg (95% CI −9.59, 0.37), NO_2_: -0.19 mmHg (95% CI −0.60, 0.22) and DBP (PM_2.5_: −0.83 mmHg (95% CI −2.93, 1.28), BC: −4.35 mmHg (95% CI −8.19, −0.50), NO_2_: −0.14 mmHg (95% CI −0.46, 0.18)). Furthermore, home-level PM_2.5_ was associated with reduced odds of having elevated BP (OR 0.75 (95% CI 0.39, 1.41)), as was BC (OR 0.35 (95% CI 0.09, 1.39)). This study showed no evidence of pro-hypertensive effect of exposure to PM_2.5_, BC, and NO_2_ pollution among schoolchildren in Accra.

## Introduction

Long-term exposure to fine particulate matter (PM_2.5_) and nitrogen dioxide (NO_2_) has been previously linked to hypertension^1–7^, the leading risk factor for global morbidity and mortality^8,9^. Emerging evidence also suggests that black carbon (BC), a component of PM and marker of combustion, may be more strongly associated with changes in blood pressure (BP) than undifferentiated PM_2.5_ mass^2,4,10^. However, the vast majority of studies were conducted among adult populations in developed countries. The current literature is sparse for children who are uniquely vulnerable to air pollution due to their still-developing cardiovascular and immune systems, higher breathing rates, and physical activity patterns^11–14^. Findings from the few studies that evaluated long-term PM_2.5_ and NO_2_ exposure and high BP in children have been inconsistent^12,15,16^, and may not be applicable to children in sub-Saharan African countries, due to differences in the spatial and temporal distribution of air pollution sources^17^, exposure levels and composition^18^, lifestyle, diet, and psychosocial stressors^2,19^.

Sub-Saharan Africa (SSA) has the highest prevalence of childhood hypertension globally, with rates as high as 25% in some countries^20,21^. This is a major public health concern given the poor rates of awareness and detection of hypertension, both in children^21^ and adults^9^, and sub-optimal access to healthcare across the region^21^. Elevated BP during childhood often tracks into adulthood and is a strong predictor for cardiometabolic disorders later in life, emphasizing the importance of identifying possible causes in early life^22,23^. In particular, there is an unmet need to evaluate the etiologic role of pervasive environmental pollution in SSA, a place where the population structure is dominated by children and nearly half of adults (≥ 25 years) have hypertension^24–27^. Given that air pollution exposure can be modified at the population level, such research may provide insights into early prevention of elevated BP among children and related morbidities in adulthood in SSA.

Greater Accra, Ghana’s largest metropolis, is experiencing high levels of ambient air pollution^28–30^, but the lack of local data at fine spatial resolution has been a hindrance to health impacts assessment^31^. Although recent studies showed that 9% of children aged 5–14 years in Accra had elevated BP^32^, the pro-hypertensive effects of urban air pollution are yet to be evaluated among this environmentally sensitive population in Ghana. Schoolchildren spend most of their day either at home or in school^2,33^, but previous studies focused on either location as an indicator of overall exposures. We therefore sought to investigate the association between exposure to ambient PM_2.5_, NO_2_, or BC at homes and/or schools and BP levels among schoolchildren recruited as part of the Accra school health and environment study (ASHES) in the Greater Accra Metropolitan Area (GAMA)^34^. We leveraged spatiotemporal land-use regression (LUR) models developed for the GAMA to estimate mean PM_2.5_, NO_2_, and BC levels at both home and school microenvironments and assessed their effects on BP.

## Methods

### Study design, location and population

Between June 2022 and May 2023, ASHES recruited 1,037 children aged 8–12 years from 90 elementary schools, comprising of both public (73%) and private (27%) schools across the GAMA. GAMA covers about 1500 km^2^ with ~ 6 million residents located inside 13 administrative districts. Most residents live within and around the most urbanized city core Accra Metropolitan Area (AMA) and the port and industrial city of Tema (TMA) to the east. The schools were selected from all 13 GAMA districts, after geocoding and mapping the locations of the ~ 700 (public) and ~ 2000 (private) primary elementary schools using data from Google Places and handheld GPS devices. Four public and one private schools in peri-urban districts, and eight or nine public and two or three private schools in the more urbanized districts were selected. This resulted in a total of 100 schools, but ten (10%) refused participation. We selected more public schools because that is where many national and local policies are implemented. Nonetheless, we included some private schools for comparison.

Students in the 4th -5th grades were invited to participate in the study, following written informed consent from school administrators and parent/guardian(s), and assent from the children themselves. Schoolchildren were eligible for inclusion if (a) they had lived at their current address for at least a year, (b) they were willing to complete children questionnaire, and (c) their parents/guardian were willing to be contacted by trained research assistants and study nurses to complete the parent survey. Of the 1,037 students, 118 (11%) were excluded because (i) parents/guardians did not complete the parent survey (*n* = 115), and (ii) residential address was outside the boundaries of the GAMA (*n* = 3), preventing the estimation of residential exposure. Thus, a total of 919 participants, comprising 691 public and 228 private school children from 90 schools were included in the final analysis.

The study was approved by the Institutional Review Boards of the University of Massachusetts Amherst and University of Ghana, Legon. Additional authorizations to conduct research within school premises were obtained from the national, regional, and district offices of the GES. All measurements were performed in accordance with relevant guidelines and regulations. Clinical trial number: not applicable.

### Blood pressure measurement

Child’s systolic and diastolic BP were measured by study nurses using Omron automated BP instrument (Model: Omron HEM7131-Z, IL, USA). Following a 5-minute rest in a sitting position, three measurements were taken on the non-dominant arm with a pediatric cuff at about 10-minute interval^14^. The average of the three measurements was used as the final BP values. Unlike adults, there was no globally unified single cut-off BP reference value for defining elevated BP in children and adolescents^35^. Thus, we calculated sex-, age-, and height-specific BP percentiles for each child. Child’s BP was classified as elevated if systolic and/or diastolic BP was at ≥ 90th percentile, following guidelines from the US National Institute of Health (NIH)^36^. The NIH guideline was adopted since there are no African specific or international guidelines for the classification of pediatric hypertension^21^.

### Air pollution exposure assessment

We estimated air pollution levels at the participants’ home and school locations using land-use regression (LUR) models developed specifically for the GAMA^37,38^, following a large-scale field measurement conducted between 2019 and 2020^28,30^. The measurement study was designed to capture temporal and spatial variations in pollutant concentrations. Thus, the field data were collected at 146 monitoring stations, involving 10 year-long sites (sampled continuously throughout the study period) and 136 week-long sites (each sampled continuously for one week). The monitoring sites were chosen based on source and land use features, including background areas (e.g., low traffic and high green space), low versus high road traffic, sparsely versus densely built-up areas, poor versus affluent and established versus emerging neighborhoods^39^ A detailed description of the measurement procedures and data can be found elsewhere^28,30^. The measurement data were then combined with geospatial and meteorological predictors to develop annual or season-specific (wet/non-Harmattan vs. dry and dusty Harmattan) LUR models at weekly and 100 m resolution across the entire GAMA. Model performances were evaluated using ten-fold cross-validation, with predictions showing good agreement with the measured data. The cross-validation Pearson R^2^ values ranged from 0.58 (mean absolute error [MAE]: 2.05) in the non-Harmattan season to 0.83 (MAE: 10.47) in the Harmattan season for PM_2.5_; from 0.79 (MAE: 0.65) to 0.88 (MAE: 10.8) for BC; and was 0.80 (root mean square error [RMSE]: 14.6) for NO_2_^37,38^. Geocoded home and school locations were then linked to the LUR model predictions, and weekly pollutant concentrations at these locations were aggregated to derive 1-year (52 weeks) and school-year (38 weeks) averages. The median (interquartile range, IQR) distance between homes and schools was 0.84 (1.44) km (range: <1–33.4 km). Consequently, estimated PM_2.5_ (*r* = 0.56), BC (*r* = 0.35), and NO_2_ (0.33) for homes and schools were moderately correlated. Similar approaches using 1-year average exposures have been employed in other pediatric studies^40–42^.

### Covariates

Several covariates (age, sex, BMI, maternal education, physical activity, socioeconomic status (SES), school type (public vs. private), household cooking fuel (dirty vs. clean), temperature, fruits, and vegetables consumption) were selected a priori based on literature review and are described below.

#### Anthropometric measures

Height of each child was measured to the nearest 0.1 cm with students standing barefoot on a portable stadiometer (model: Seca 216, Hamburg, Germany) and weight to the nearest 0.1 kg using a calibrated digital weighing scale (model: Seca 813, Hamburg, Germany) without shoes and bulky clothing. We then calculated body mass index (BMI, kg/m^2^) as the weight (kg) divided by the squared height (m^2^). BMI-for-age z-scores were calculated according to the World Health Organization (WHO) child growth standards and categorized into underweight, normal, overweight, and obese (World Health Organization,^43^.

#### Demographic, lifestyle, and weather factors

Structured questionnaires were used to gather extensive data about the child and other household characteristics. The pre-tested survey questions were administered by trained field staff and managed using REDCap electronic data capture tools hosted at the University of Massachusetts Chan Medical School^44,45^. Demographic and lifestyle information were completed by the children and their parents. Date of birth was used to estimate completed age at the time of the BP measurements. Maternal education was used as a proxy for individual socio-economic status (SES) and categorized as low (primary school or lower), medium (secondary school), or high (college or university). Community-level SES was estimated from expenditure data collected by the Ghana living standards surveys and assessed as the median log equivalized household consumption (Ghanaian Cedi) for the census enumeration area (EA: smallest census unit) containing subject’s residence; details can be found elsewhere^46^. Home EA SES was classified as below or above (≥) the median EA SES. Information on primary household cooking fuel was provided by parents and categorized as clean (electricity, LPG) or dirty (charcoal, wood, kerosene).

Weekly physical activity included all forms of outdoor exercise and was derived from children’s self-reported frequency of exercise (days) over the preceding week and defined as low (≤ 2 days/week), medium (3–4 days/week), and high (> 4 days/week). Children were also asked to indicate “whether or not they ate (a) fruits (b) vegetables in the past 24 hours”. Minute-level ambient air temperature data was collected at a peri-urban background location and averaged to daily values to correspond to BP measurement date for each child.

### Statistical analysis

Descriptive statistics were used to summarize the characteristics of the participants, BP measures, and the air pollution data. BC, a component of PM_2.5_, was measured as the optical absorption coefficient, which quantifies the amount of light absorbed by particles collected on the PM_2.5_ filters. For interpretability, BC was scaled per 10 × 10^− 5^m^−^, representing a realistic range of exposure. Qualitative and quantitative variables were reported as numbers (%) and mean (standard deviation (SD)), respectively. Chi-square tests were used to compare the prevalences of elevated BP across participant characteristics, while t-test and analysis of variance (ANOVA) were used to examine differences in mean values of continuous covariates and BP outcomes.

Linear mixed-effects models were used to evaluate the associations between air pollutants and mean systolic and diastolic BP, while logistic regression models were used to estimate the odds of having elevated BP in relation to air pollutant exposures. Each pollutant was modeled separately in single-pollutant models, with the associations assessed for school-level, home-level, and the average of school- and home-level exposures. Separate models were developed for annual PM_2.5_, BC, and NO_2_ levels at home, school, as well as the average pollutant level across both locations. In sensitivity analyses, we also examined two-pollutant models by mutually adjusting for PM_2.5_ and NO_2_ in each model. Although elementary schools in Ghana are not zoned by district of residence, 58% of children in this study attended schools located within 1 km of their residential address. Thus, to account for potential clustering within schools or homes in the same neighborhoods, home and school models included random intercepts for residential and school municipality, respectively. The models were specified as follows:

$$\:SBP\:or\:DBP=\:{\beta\:}_{o}+\:{\beta\:}_{1}{X}_{1}+\:{\beta\:}_{i}{Z}_{i}+\lambda\:+\:\epsilon\:\:$$

$$\:logit\left[Probability\:\right(\mathrm{E}\mathrm{l}\mathrm{e}\mathrm{v}\mathrm{a}\mathrm{t}\mathrm{e}\mathrm{d}\:\mathrm{B}\mathrm{P}\left)\right]=\:{\beta\:}_{o}+{\beta\:}_{1}{X}_{1}+{\beta\:}_{i}{Z}_{i}+\lambda\:+\:\epsilon\:\:$$

- X = annual ambient air pollution either at home, school, or average of both locations.
- Z = a vector of covariates.
- $\:\lambda\:$ = random intercept (district ID and/or school ID).
- ε = error term.

Previous literature was used to identify potential confounding variables a priori for inclusion in multiple regression models. Models were adjusted for age (continuous), sex, BMI, physical activity, maternal education, neighborhood SES, school category (public vs. private), cooking fuel (clean vs. dirty), temperature, fruits, and vegetables consumption as fixed effects.

All analyses were performed using R statistical package (R Core Team,^47^, version 4.3.2 and a two-tailed p-value of < 0.05 was considered statistically significant. Estimates [with 95% confidence intervals (CIs)] are presented for 10 unit increases in pollutant concentrations.

## Results

### Characteristics of the participants

A total of 919 schoolchildren (60% females; aged 10.7 [SD: 1.2]) were included in this analysis (Table 1). The average BMI was 18.6 (3.8) kg/m^2^ with 30% of the schoolchildren classified as either overweight or obese (based on the WHO’s BMI-for-age z-scores). Most mothers of the participants had secondary or higher education (60%), and the majority (72%) lived in households that used LPG or electricity as their primary cooking fuel. These estimates are similar to the distribution of women education and household fuel use reported in the recent national census data for the Greater Accra Region.

### BP parameters

The mean systolic and diastolic BP of all participants were 107.2 (9.7) and 69.1 (7.1) mmHg, respectively. Systolic BP was higher in older (≥ 10-year-olds) children (108 vs. 105 mmHg), and those classified as overweight/obese had both higher systolic (109 vs. 106 mmHg) and diastolic (70 vs. 68 mmHg) BP values at p-value < 0.05. The overall prevalence of elevated BP was 33%, with similar rates for pre-hypertension (15%) and hypertension (18%). Additionally, the prevalence of elevated BP was higher in children living in neighborhoods with lower EA SES (36% vs. 30%), overweight children (41% vs. 30%), and those attending public schools compared to their counterparts in private schools (35% vs. 28%) at p-value < 0.05.

### Air pollution exposure

**Fig. 1:**
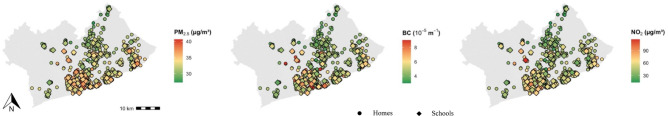
The map of GAMA and the distribution of annual PM_2.5_, BC, and NO_2_ values across participants geocoded home and school locations. Estimates of pollutant concentrations are based on the recently developed land use regression models.

Figure 1 shows the map of GAMA and the distribution of LUR-predicted annual PM_2.5_, BC, and NO_2_ concentrations based on participants geocoded residential and school locations. Overall, predicted annual PM_2.5_ exposure levels for homes (mean: 34, range: 27–41 µg/m^3^) and schools (mean: 30, range: 26–35 µg/m^3^) of study participants were similar, but are 5–8 times higher than the current WHO guideline of 5 µg/m^3^ (Table 2). The mean annual BC (5 ⋅ 10^− 5^m^− 1^ or 8.4 µg/m^3^) estimated for both locations was 40% higher than the maximum value (3 ⋅ 10^− 5^m^− 1^ or 5.1 µg/m^3^) reported in WHO’s “good practice statement for BC”. In addition, 30% of participants were exposed to home-level PM_2.5_ concentrations that exceeded the WHO’s IT-1 (35 µg/m^3^), while all participants had BC levels higher than 5.1 µg/m^3^. PM_2.5_ estimates for homes and schools were more correlated (*r* = 0.56) than for BC (*r* = 0.35) (Table 2).There were similarities between annual NO_2_ exposure levels for homes (mean: 55, range: 13–109) and schools (mean: 54, range: 23–117), both of which were above the current WHO annual guideline of 10 µg/m^3^.

**Table 1:** Characteristics of the study population (N = 919).

Variables	Mean ± SD	Range
Age (year)*	10.8 ± 1.2	8–12
Weight (kg)	35.1 ± 9.0	18.1–91.7
Height (cm)	137.9 ± 10.9	101.0–167.0
Body mass index (kg/m^2^)	18.6 ± 3.8	11.9–37.9
Systolic blood pressure (mmHg)	107.2 ± 9.7	80.0–149.0
Diastolic blood pressure (mmHg)	69.1 ± 7.1	46.7–101.3

	n	%
Male	369	40.1
Blood pressure category		
Normal	616	67.0
Pre-hypertension	140	15.3
Hypertension	163	17.7
Physical exercise*		
≤ 2 times per week	361	39.3
3–4 times per week	338	36.8
> 4 times per week	220	23.9
Body mass index category*		
Underweight	30	3.3
Normal weight	611	66.5
Overweight	174	18.9
Obesity	104	11.3
Maternal education		
Primary school or lower	365	39.7
Secondary school	378	41.1
College or University	176	19.2
Cooking fuel used at home		
Clean (LPG, Electricity)	661	72.1
Dirty (Charcoal, Wood)	256	27.9
School category*		
Public	691	75.2
Private	228	24.8
EA SES (< median)	459	49.9
Vegetable intake in last 24 h (Yes)	272	29.6
Fruit intake in last 24 h (Yes)	257	27.9

* Systolic BP, diastolic BP or elevated BP differ between groups at *p* < 0.05.

**Table 2:** Summary of annual average concentrations of air pollutants by location.

Air pollutants	Mean (SD)	Median (IQR)	Range	r
PM_2.5_ (µg/m^3^)				0.56
Home	34 (2)	33 (3)	27–41	
School	30 (2)	30 (3)	26–35	
BC (1⋅ 10^− 5^m^− 1^)				0.35
Home	5.4 (1)	5.2 (1)	3.1–15	
School	5.1 (1)	5.0 (1)	3.3–8.9	
NO_2_ (µg/m^3^)				0.33
Home	55 (15)	56 (19)	13–109	
School	54 (15)	54 (19)	23–117	

### Associations between PM_2.5_, BC, NO_2_ and blood pressure

Table 3 shows the estimates for the associations between air pollution exposures at home, school, and home/school locations and BP parameters. Overall, we did not observe any positive associations. For instance, a 10 µg/m^3^ increase in annual predicted home-level PM_2.5_ was associated with a 0.70 mmHg decrease in systolic BP (95% CI: -3.34, 1.94) and a 0.83 mmHg decrease in diastolic BP (95% CI: -2.93, 1.28). Similarly, at school locations, PM_2.5_ levels were associated with 2.01 mmHg (95% CI: -5.24, 1.22) and 2.49 mmHg (95% CI: -5.06, 0.08) lower systolic and diastolic BP levels, respectively. However, confidence intervals across all models included the null. Inverse associations were also observed for every 10 ×10^− 5^m^− 1^ increment in annual predicted BC levels, both at home (-4.61 mmHg (95% CI: -9.59, 0.37) for systolic BP and − 4.35 mmHg (95% CI: -8.19, -0.50) for diastolic BP) and at school locations (-8.01 mmHg (95% CI: -14.43, -1.59) for systolic BP and − 6.81 mmHg (95% CI: -11.86, -1.76) for diastolic BP. Additionally, each 10 µg/m^3^ increase in annual predicted NO_2_ levels was associated with lower BP, both at home (systolic BP: -0.19 mmHg (95% CI: -0.60, 0.22); diastolic BP: -0.14 mmHg (95% CI: -0.46, 0.18) and at school locations (systolic BP: -0.40 mmHg (95% CI: -0.81, 0.02); diastolic BP: -0.43 mmHg (95% CI: -0.76, -0.10). In logistic regression analyses, predicted PM_2.5_ was associated with decreased odds of having elevated BP (OR_Home_: 0.75, 95% CI: 0.39, 1.41; OR_School_: 0.90, 95% CI: 0.41, 2.01), as was BC (OR_Home_: 0.35, 95% CI: 0.09, 1.39; OR_School_: 0.18, 95% CI: 0.04, 0.84), and NO_2_ exposure (OR_Home_: 0.98, 95% CI: 0.89, 1.08; OR_School_: 0.93, 95% CI: 0.84, 1.03). Lastly, we observed negative and/or null associations with BP outcomes for every 10-unit increase in average home- and school-level PM_2.5_, BC, and NO_2_ exposures (Table 3). Results from two-pollutant models were similar to single-pollutant models (results not shown). To assess potential multicollinearity, we examined generalized variance inflation factors (GVIF); adjusted GVIF values were all < 2, indicating low multicollinearity across all models.

**Table 3:** Associations between annual predicted air pollution and blood pressure parameters.

Exposure	Coefficient (95% CI)		Odds ratio (95% CI)
	Systolic BP (mmHg)	Diastolic BP (mmHg)	Elevated BP
PM_2.5_ (µg/m^3^)			
Home ^a^	-0.70 (-3.34,1.94)	-0.83 (-2.93, 1.28)	0.75 (0.39, 1.41)
School ^b^	-2.01 (-5.24,1.22)	-2.49 (-5.06, 0.08)	0.90 (0.41, 2.01)
Home and School ^c^	-1.63 (-4.97, 1.70)	-1.97 (-4.73, 0.79)	0.74 (0.33, 1.67)
BC (1⋅ 10^− 5^m^− 1^)			
Home ^a^	-4.61 (-9.59, 0.37)	-4.35 (-8.19, -0.50) *	0.35 (0.09, 1.39)
School ^b^	-8.01 (-14.43, -1.59) *	-6.81 (-11.86, -1.76) *	0.18 (0.04, 0.84) *
Home and School ^c^	-9.07 (-15.96, -2.17) *	-8.04 (-13.62, -2.46) *	0.13 (0.02, 0.72) *
NO_2_ (µg/m^3^)			
Home ^a^	-0.19 (-0.60, 0.22)	-0.14 (-0.46, 0.18)	0.98 (0.89, 1.08)
School ^b^	-0.40 (-0.81, 0.02)	-0.43 (-0.76, -0.10) *	0.93 (0.84, 1.03)
Home and School ^c^	-0.46 (-0.98, 0.06)	-0.43 (-0.85, -0.01) *	0.93 (0.82, 1.05)

Effect estimates were scaled to 10 µg/m^3^ or 10×10^− 5^m^− 1^ increment in PM_2.5_ and BC concentrations, respectively.

CI: confidence interval; BP: Blood pressure.

Random effects: ^a^ Residential district, ^b^ School district, ^c^ Residential and school district.

*P-value < 0.05.

Models adjusted for age, sex, BMI, maternal education, physical activity, home EA SES, school category, cooking fuel, temperature, fruits, and vegetables consumption.

## Discussion

Air pollution has been consistently linked to elevated BP among adults, but evidence of this association in children and adolescents is limited, especially in SSA. To our knowledge this study is only the second in SSA to investigate the effect of long-term ambient air pollution on BP among children. We leveraged unique local LUR models to estimate annual home- and school-level PM_2.5_, BC, and NO_2_ exposures and measured BP of 919 children from 90 elementary schools distributed across all municipalities in the GAMA. Every child’s PM_2.5_ exposure at both home and school was above 5 µg/m^3^, the WHO annual guideline, including 30% of the children whose exposure at home exceeded the least stringent interim guideline (IT-3: 35 µg/m^3^). Notwithstanding the elevated ambient air pollution exposure in this population, we found no evidence of positive associations between long-term exposure to PM_2.5_, BC, or NO_2_ and BP outcomes among these children. Across home, school, and combined exposure metrics, most estimates were null or inversely associated with BP, and most confidence intervals included the null. Although some inverse associations, particularly for BC and NO_2_ at school locations, reached statistical significance, these patterns were not consistent across pollutants or exposure settings. Logistic regression results similarly indicated no increased odds of elevated BP with higher pollutant exposures, and findings were robust to multi-pollutant adjustment, with low multicollinearity observed.

Despite the lack of positive associations with air pollution, the burden of elevated BP in this population was substantial. Higher BP levels and a greater prevalence of elevated BP were observed among older children, those with overweight or obesity, children living in lower socioeconomic neighborhoods, and those attending public schools. Taken together, these findings suggest that while ambient air pollution was not associated with increased BP in this cohort, cardiometabolic risk factors and social determinants may play a more prominent role in shaping BP patterns among children in this setting.

The prevalence of elevated BP and hypertension in our study were three times higher than previously reported for Greater Accra^32^, but were within the range (0.2–38.9%) reported in recent systematic reviews which concluded that SSA has the highest pooled prevalence (7%) of childhood hypertension^21,26^. Still, it is important to note that, we classified elevated BP using sex-, age-, and height-specific percentiles based on U.S. NIH guidelines, due to the absence of locally derived or internationally harmonized reference standards for children. While this approach is consistent with prior studies and current recommendations where local references are unavailable, these percentiles may not be fully generalizable to urban Ghanaian children and could have led to some misclassification of BP status. Additionally, BP measurements were obtained under field conditions, where factors such as ambient heat, environmental noise, and children’s activity levels or excitement may have influenced readings. These conditions could have introduced measurement variability and potentially resulted in transiently elevated BP values. Consistent with existing literature in Ghana^32,48^ and globally^49–52^ though, we found that BP levels significantly increased with age and BMI, emphasizing the need for targeted social interventions (e.g., school- and community-based health and physical education programs) that promote culturally tailored healthy lifestyles^23,32,51^.

While the estimated ambient PM_2.5_, BC, and NO_2_ exposures of schoolchildren in the GAMA far exceeded international guidelines, they were not associated with increased systolic, diastolic, or elevated BP. Our findings are consistent with the heterogeneous findings reported in pediatric populations, including those reported in American^11^, German^53^, and South African^54^ children, but inconsistent with studies from elsewhere^15,40,41,55,56^. The heterogeneity could be caused by variations in the methodology, participant characteristics (e.g., pubertal status, psychosocial stressors), and exposure levels and patterns^16,57^.

The observed unexpected inverse or null associations with pollutants should be interpreted cautiously, as several biological, methodological, and contextual factors, particularly in low- and middle-income settings, may underlie these findings. First, residual confounding by socioeconomic and environmental characteristics may have influenced the observed associations, particularly in urban SSA settings where pollution patterns may not align with deprivation in the same way as in high-income countries. Second, exposure misclassification may have attenuated true associations. Despite the use of locally developed LUR models to provide improved spatial resolution, they may not fully capture individual-level exposures across diverse microenvironments (e.g. indoor, household, and in-transit) and time-activity patterns. Third, residual confounding from unmeasured factors remains possible despite adjustment for a wide range of covariates. Unmeasured factors such as nutrition (e.g. sodium intake or fried food consumption), stress and infection burden may also mask or counteract potential effects of air pollution on BP^58^. Fourth, BP measurement variability under field conditions may have introduced random error, further biasing results toward the null. Finally, while inclusion of variables that lie on the causal pathway (e.g., BMI or physical activity) or act as colliders could have attenuated or reversed observed associations^59^, our results remain essentially unchanged when BMI and measures of physical activity were excluded from the models.

Taken together, these findings likely reflect a combination of methodological and contextual factors rather than a true protective effect, underscoring the need for longitudinal studies with refined exposure assessment in similar settings.

### Strengths and limitations

This is the first citywide study on the pro-hypertensive effects of children’s long-term exposure to PM_2.5_ and its combustion-related component BC, and NO_2_ in Ghana and largest in SSA. We used geocoded residential and school locations data to estimate exposure across the full range of pollutant concentrations in Accra using advanced modeling methods. Since Ghana does not utilize residence-based school assignment policy, this approach allowed us to separately evaluate the associations in both microenvironments. In addition, we improved upon the previous study in SSA, by adjusting for other important covariates such as diet, physical activity, as well as indicators of household air pollution, greenness, road traffic noise, and economic deprivation that could affect validity of the results due to residual confounding.

However, there are also some limitations to consider. First, due to the cross-sectional nature of our study, we are unable to establish causal relationships between air pollutant exposures and BP parameters, and therefore, the observed associations should be considered exploratory. Second, our BP parameters measured on a single day could not precisely determine each participant’s true BP status, which could lead to misclassification of BP status and influence the magnitude of estimated associations. Third, although our exposure estimates were derived from validated local LUR models based on 2019–2020 monitoring data, BP measurements were collected between 2022 and 2023. While data from ten fixed monitoring stations suggested that spatial patterns of pollutants remained relatively stable over time, we cannot rule out temporal misalignment between exposure estimates and BP measurements. This lag may have introduced additional exposure uncertainty, leading to non-differential misclassification and, consequently, potential attenuation of the observed association. Moreover, our exposure metrics captured relatively recent chronic exposures rather than long-term, cumulative exposures over multiple years. As a result, they may not adequately represent lifetime exposure windows that are more relevant for the development and progression of sustained changes in blood pressure. Fourth, we excluded 11% of consented participants due to incomplete information, which could impact the generalizability of results. However, the characteristics of these participants did not differ from those with complete data. Fifth, despite the detailed characterization of potential confounders, most were self-reported and residual confounding from unmeasured factors remains a possibility. Overall, these results are exploratory, and longitudinal studies with repeated BP measurements are needed to confirm our findings and explore the temporal relationship and seasonal trends between air pollution exposure and children’s BP.

## Conclusion

This study contributed to the scant literature on the effects of chronic air pollution exposure among children in SSA, a group that has been underrepresented in research. The findings bring attention to the increasing prevalence of elevated BP in children and strategies to reverse this trend given that BP tracks from childhood to adulthood. We provided evidence of children’s exposure to poor air quality in the environments where they spend most of their time learning and playing. Though, our findings do not support adverse effects on children’s BP, existing knowledge on physiological responses to chronic air pollution exposure compels stringent emission control and regulatory actions to create healthier learning and living environments for children in the GAMA.

## Data Availability

Environmental measurement data is available through the open-source platform, Zenodo, De-identified health data can be made available to investigators with projects that fall within the overall aim of ASHES. Those interested in gaining access to the data should contact Raphael E Arku [rarku@umass.edu].

## References

[1] Arku, R. E. et al. Long-term exposure to outdoor and household air pollution and blood pressure in the Prospective Urban and Rural Epidemiological (PURE) Study HHS public access. *Environ. Pollut.* **262**, 114197. 10.1016/j.envpol.2020.114197 (2020). 32146361 10.1016/j.envpol.2020.114197 PMC7767575

[2] de Bont, J. et al. Ambient air pollution and cardiovascular diseases: An umbrella review of systematic reviews and meta-analyses. *J. Intern. Med.* **291**(6), 779–800. 10.1111/joim.13467 (2022). 35138681 10.1111/joim.13467 PMC9310863

[3] Cai, Y. et al. Associations of short-term and long-term exposure to ambient air pollutants with hypertension: A systematic review and meta-analysis. *Hypertension* **68**(1), 62–70. 10.1161/HYPERTENSIONAHA.116.07218/-/DC1 (2016). 27245182 10.1161/HYPERTENSIONAHA.116.07218

[4] Giorgini, P. et al. Air pollution exposure and blood pressure: an updated review of the literature. *Curr. Pharm. Des.* **22**(1), 28–51. 10.2174/1381612822666151109111712 (2015). 10.2174/1381612822666151109111712 26548310

[5] Rajagopalan, S., Al-Kindi, S. G. & Brook, R. D. Air pollution and cardiovascular disease: JACC State-of-the-Art Review. *J. Am. Coll. Cardiol.* **72**(17), 2054–2070. 10.1016/j.jacc.2018.07.099 (2018). 30336830 10.1016/j.jacc.2018.07.099

[6] Yang, B. Y. et al. Global association between ambient air pollution and blood pressure: A systematic review and meta-analysis. *Environ. Pollut.* **235**, 576–588. 10.1016/j.envpol.2018.01.001 (2018). 29331891 10.1016/j.envpol.2018.01.001

[7] Yang, L., Magnussen, C. G., Yang, L., Bovet, P. & Xi, B. Elevated blood pressure in childhood or adolescence and cardiovascular outcomes in adulthood: A systematic review. *Hypertension*10.1161/HYPERTENSIONAHA.119.14168/FORMAT/EPUB (2020). 32114851 10.1161/HYPERTENSIONAHA.119.14168

[8] Schutte, A. E., Srinivasapura, N. & Mohan, S. Hypertension in low-and middle-income countries HHS Public Access. *Circ. Res.* **128**(7), 808–826. 10.1161/CIRCRESAHA.120.318729 (2021). 33793340 10.1161/CIRCRESAHA.120.318729 PMC8091106

[9] Zhou, B., Perel, P., Mensah, G. A. & Ezzati, M. Global epidemiology, health burden and effective interventions for elevated blood pressure and hypertension. *Nat. Rev. Cardiol.* **18**(11), 785–802. 10.1038/s41569-021-00559-8 (2021). 34050340 10.1038/s41569-021-00559-8 PMC8162166

[10] Magalhaes, S., Baumgartner, J. & Weichenthal, S. Impacts of exposure to black carbon, elemental carbon, and ultrafine particles from indoor and outdoor sources on blood pressure in adults: A review of epidemiological evidence. *Environ. Res.* **161**, 345–353. 10.1016/j.envres.2017.11.030 (2018). 29195183 10.1016/j.envres.2017.11.030

[11] Liu, C. et al. The associations between traffic-related air pollution and noise with blood pressure in children: Results from the GINIplus and LISAplus studies. *Int. J. Hyg. Environ. Health* **217**(4–5), 499–505. 10.1016/j.ijheh.2013.09.008 (2014). 24183515 10.1016/j.ijheh.2013.09.008

[12] Qin, P. et al. Long-term association of ambient air pollution and hypertension in adults and in children: A systematic review and meta-analysis. *Sci. Total Environ.* **796**, 148620. 10.1016/J.SCITOTENV.2021.148620 (2021). 34274662 10.1016/j.scitotenv.2021.148620

[13] Tandon, S. et al. Association of ambient air pollution with blood pressure in adolescence: a systematic-review and meta-analysis. Curr. Prob. Cardiol. 10.17605/OSF.IO/96G5Q (2023). 10.1016/j.cpcardiol.2022.101460 36265590

[14] Zhang, J. et al. Air pollution-associated blood pressure may be modified by diet among children in Guangzhou, China. *J. Hypertens.* **38**(11), 2215–2222. 10.1097/HJH.0000000000002521 (2020). 32649627 10.1097/HJH.0000000000002521

[15] Huang, M. et al. Effects of ambient air pollution on blood pressure among children and adolescents: A systematic review and meta-analysis. *J. Am. Heart Assoc.*10.1161/JAHA.120.017734 (2021). 33942625 10.1161/JAHA.120.017734 PMC8200690

[16] Yan, M. et al. Associations between ambient air pollutants and blood pressure among children and adolescents: A systemic review and meta-analysis. *Sci. Total Environ.* **785**(22), 147279. 10.1016/j.scitotenv.2021.147279 (2021). 33940406 10.1016/j.scitotenv.2021.147279

[17] Katoto, P. D. M. C. et al. Ambient air pollution and health in Sub-Saharan Africa: Current evidence, perspectives and a call to action. *Environ. Res.* **173**, 174–188. 10.1016/j.envres.2019.03.029 (2019). 30913485 10.1016/j.envres.2019.03.029

[18] Coker, E. & Kizito, S. A narrative review on the human health effects of ambient air pollution in Sub-Saharan Africa: An urgent need for health effects studies. *Int. J. Environ. Res. Public Health*10.3390/ijerph15030427 (2018). 29494501 10.3390/ijerph15030427 PMC5876972

[19] Ewald, D. R. & Haldeman, L. A. Risk factors in adolescent hypertension. *Glob. Pediatric Health*10.1177/2333794x15625159 (2016). 10.1177/2333794X15625159 PMC4784559 27335997

[20] Amadi, O. et al. Hypertension in children: Could the prevalence be on the increase?. *Niger. Med. J.* **60**(5), 262. 10.4103/nmj.nmj_51_19 (2019). 31844356 10.4103/nmj.NMJ_51_19 PMC6900897

[21] Crouch, S. H. et al. Paediatric hypertension in Africa: a systematic review and meta-analysis. *eClinicalMedicine.* **43**, 101229. 10.1016/j.eclinm.2021.101229 (2022). 34917909 10.1016/j.eclinm.2021.101229 PMC8665406

[22] Chen, X. & Wang, Y. Tracking of blood pressure from childhood to adulthood: A systematic review and meta-regression analysis. *Circulation* **117**(25), 3171–3180. 10.1161/CIRCULATIONAHA.107.730366 (2008). 18559702 10.1161/CIRCULATIONAHA.107.730366 PMC3568631

[23] Liang, X., Xiao, L., Luo, Y. & Xu, J. Prevalence and risk factors of childhood hypertension from birth through childhood: A retrospective cohort study. *J. Hum. Hypertens.* **34**(2), 151–164. 10.1038/s41371-019-0282-z (2020). 31666662 10.1038/s41371-019-0282-z

[24] Ferdinand, K. C. Uncontrolled hypertension in Sub-Saharan Africa: Now is the time to address a looming crisis. *J. Clin. Hypertens.* **22**, 2111–2113. 10.1111/jch.14046 (2020). 10.1111/jch.14046 PMC8030060 32951284

[25] Mills, K. T. et al. Global disparities of hypertension prevalence and control: A systematic analysis of population-based studies from 90 countries. *Circulation* **134**, 441–450. 10.1161/CIRCULATIONAHA.115.018912 (2016). 27502908 10.1161/CIRCULATIONAHA.115.018912 PMC4979614

[26] Noubiap, J. J. et al. Prevalence of elevated blood pressure in children and adolescents in Africa: A systematic review and meta-analysis. *Lancet Public Health* **2**(8), e375–e386. 10.1016/S2468-2667(17)30123-8 (2017). 29253478 10.1016/S2468-2667(17)30123-8

[27] UNICEF, U. N. I. C. E. F. *Children in Africa: Key statistics on child survival and population - UNICEF DATA*. (Accessed 18 January 2023). https://data.unicef.org/resources/children-in-africa-child-survival-brochure/. (2017).

[28] Alli, A. S. et al. Spatial-temporal patterns of ambient fine particulate matter (PM 2.5) and black carbon (BC) pollution in Accra. *Environ. Res. Lett.* **16**(7), 1–13. 10.1088/1748-9326/ac074a (2021). 10.1088/1748-9326/ac074a PMC8227509 34239599

[29] Dionisio, K. L. et al. Air pollution in Accra neighborhoods: Spatial, socioeconomic, and temporal patterns. *Environ. Sci. Technol.* **44**(7), 2270–2276. 10.1021/es903276s (2010). 20205383 10.1021/es903276s

[30] Wang, J. et al. Nitrogen oxides (NO and NO2) pollution in the Accra metropolis: Spatiotemporal patterns and the role of meteorology. *Sci. Total Environ.* **803**(2), 149931. 10.1016/j.scitotenv.2021.149931 (2022). 34487903 10.1016/j.scitotenv.2021.149931 PMC7611659

[31] Yawson, A. E. et al. Non-communicable diseases among children in Ghana: Health and social concerns of parent/caregivers. *Afr. Health Sci.* **16**(2), 378–388. 10.4314/ahs.v16i2.6 (2016). 27605953 10.4314/ahs.v16i2.6 PMC4994565

[32] Afaa, T. J., Amegan-aho, K. H., Asante, Y. A. & Rodrigues, O. P. Prevalence of high blood pressure, overweight, and obesity in Ghanaian school children. *Health Sci. Investig.* **3**(2), 360–364. 10.46829/hsijournal.2022.12.3.2.360-364 (2022).

[33] Arku, R. E. et al. Personal particulate matter exposures and locations of students in four neighborhoods in Accra, Ghana. *J. Expo. Sci. Environ. Epidemiol.* **25**(6), 557–566. 10.1038/jes.2014.56 (2015). 25160763 10.1038/jes.2014.56

[34] Lange, C. L. et al. The Accra school health and environment study (ASHES): A study of the urban environment and child health and development in Accra. *Environ. Res. Commun.* **8**(3), 035012. 10.1088/2515-7620/ae4a78 (2026). 42180412 10.1088/2515-7620/ae4a78 PMC13193033

[35] Xi, B. et al. Establishing international blood pressure references among nonoverweight children and adolescents aged 6 to 17 years. *Circulation* **133**(4), 398–408. 10.1161/CIRCULATIONAHA.115.017936 (2016). 26671979 10.1161/CIRCULATIONAHA.115.017936 PMC4729639

[36] Falkner, B. & Daniels, S. R. Summary of the fourth report on the diagnosis, evaluation, and treatment of high blood pressure in children and adolescents. *Hypertension*10.1161/01.HYP.0000143545.54637.af (2004). 15353515 10.1161/01.HYP.0000143545.54637.af

[37] Alli, A. S. et al. High-resolution patterns and inequalities in ambient fine particle mass (PM2.5) and black carbon (BC) in the Greater Accra Metropolis Ghana. *Sci. Total Environ.*10.1016/J.SCITOTENV.2023.162582 (2023). 36870487 10.1016/j.scitotenv.2023.162582 PMC10131145

[38] Wang, J. et al. Inequalities in urban air pollution in sub-Saharan Africa: An empirical modeling of ambient no and no2 concentrations in Accra, Ghana. *Environ. Res. Lett.* **19**(3), 034036. 10.1088/1748-9326/ad2892 (2024). 38419692 10.1088/1748-9326/ad2892 PMC10897512

[39] Clark, S. N. et al. High-resolution spatiotemporal measurement of air and environmental noise pollution in Sub-Saharan African cities: Pathways to Equitable Health Cities Study protocol for Accra, Ghana. *BMJ Open* **10**, 35798. 10.1136/bmjopen-2019-035798 (2020). 10.1136/bmjopen-2019-035798 PMC7440835 32819940

[40] Bilenko, N. et al. Associations between particulate matter composition and childhood blood pressure - The PIAMA study. *Environ. Int.* **84**(2), 1–6. 10.1016/j.envint.2015.07.010 (2015). 26186643 10.1016/j.envint.2015.07.010

[41] Li, J. et al. Long-term effects of PM2.5 components on blood pressure and hypertension in Chinese children and adolescents. *Environ. Int.* **161**, 107134. 10.1016/j.envint.2022.107134 (2022). 35180672 10.1016/j.envint.2022.107134

[42] Wang, X. et al. Association of school residential PM2.5 with childhood high blood pressure: Results from an observational study in 6 cities in China. *Int. J. Environ. Res. Public Health* **16**(14), 2515. 10.3390/ijerph16142515 (2019). 31337125 10.3390/ijerph16142515 PMC6678215

[43] World Health Organization. *Growth reference indicators. BMI-for-age (5–19 years)*. (Accessed 23 January 2023). https://www.who.int/tools/growth-reference-data-for-5to19-years/indicators/bmi-for-age. (2007).

[44] Harris, P. A. et al. The REDCap consortium: Building an international community of software platform partners. *J. Biomed. Inform.* **95**, 103208. 10.1016/J.JBI.2019.103208 (2019). 31078660 10.1016/j.jbi.2019.103208 PMC7254481

[45] Harris, P. A. et al. Research electronic data capture (REDCap)—A metadata-driven methodology and workflow process for providing translational research informatics support. *J. Biomed. Inform.* **42**(2), 377–381. 10.1016/J.JBI.2008.08.010 (2009). 18929686 10.1016/j.jbi.2008.08.010 PMC2700030

[46] Cavanaugh, A. C. et al. Strangers in a strange land: Mapping household and neighbourhood associations with improved wellbeing outcomes in Accra, Ghana. *Cities* **143**, 104584. 10.1016/J.CITIES.2023.104584 (2023). 37829151 10.1016/j.cities.2023.104584 PMC7615188

[47] R Core Team. *The R Project for Statistical Computing, Vienna, Austria*, *A language and environment for statistical computing. R Foundation for Statistical Computing, Vienna, Austria.* (Accessed 12 March 2020). https://www.r-project.org/. (2021).

[48] Agyemang, C., Redekop, W. K., Owusu-Dabo, E. & Bruijnzeels, M. A. Blood pressure patterns in rural, semi-urban and urban children in the Ashanti region of Ghana, West Africa. *BMC Public Health* **5**, 1–7. 10.1186/1471-2458-5-114 (2005). 16262905 10.1186/1471-2458-5-114 PMC1289286

[49] Brady, T. M., Schmidts, M., Kupferman, J. C. & Chishti, A. S. Obesity-related hypertension in children. *Front. Pediatr.*10.3389/fped.2017.00197 (2017). 28993801 10.3389/fped.2017.00197 PMC5622310

[50] Rutigliano, I. et al. Obesity-related hypertension in pediatrics, the impact of American Academy of Pediatrics Guidelines. *Nutrients*10.3390/nu13082586 (2021). 34444745 10.3390/nu13082586 PMC8398436

[51] Torrance, B., Mcguire, A., Lewanczuk, R. & Mcgavock, J. Overweight, physical activity and high blood pressure in children: A review of the literature. *Vasc. Health Risk Manag.* **3**(1), 139–149 (2007). 17583184 PMC1994042

[52] Zhang, Y. X. & Wang, S. R. The relationship of body mass index distribution to relatively high blood pressure among children and adolescents in Shandong, China. *Ann. Hum. Biol.* **38**(5), 630–634. 10.3109/03014460.2011.594453 (2011). 21745152 10.3109/03014460.2011.594453

[53] Mann, J. K. et al. Traffic-related air pollution is associated with glucose dysregulation, blood pressure, and oxidative stress in children. *Environ. Res.* **195**, 110870. 10.1016/j.envres.2021.110870 (2021). 33587949 10.1016/j.envres.2021.110870 PMC8520413

[54] Chungag, A., Engwa, G. A., Sewani-Rusike, C. R. & Nkeh-Chungag, B. N. Effect of seasonal variation on the relationship of indoor air particulate matter with measures of obesity and blood pressure in children. *J. Health Pollut.* **11**(30), 1–11. 10.5696/2156-9614-11.30.210610 (2021). 10.5696/2156-9614-11.30.210610 PMC8276733 34267997

[55] Wu, Q. Z. et al. Ambient airborne particulates of diameter ≤1 μm, a leading contributor to the association between ambient airborne particulates of diameter ≤2.5 μm and children’s blood pressure. *Hypertension*10.1161/HYPERTENSIONAHA.119.13504 (2019). 31838909 10.1161/HYPERTENSIONAHA.119.13504

[56] Zhang, Z. et al. Exposure to ambient particulate matter air pollution, blood pressure and hypertension in children and adolescents: A national cross-sectional study in China. *Environ. Int.* **128**, 103–108. 10.1016/j.envint.2019.04.036 (2019). 31035113 10.1016/j.envint.2019.04.036

[57] Pedersen, M. et al. Associations between ambient air pollution and noise from road traffic with blood pressure and insulin resistance in children from Denmark. *Environ. Epidemiol.* **3**(5), 1–7. 10.1097/EE9.0000000000000069 (2019). 10.1097/EE9.0000000000000069 PMC7939405 33778342

[58] Leyvraz, M. et al. Sodium intake and blood pressure in children and adolescents: a systematic review and meta-analysis of experimental and observational studies. *Int. J. Epidemiol.* **47**(6), 1796–1810. 10.1093/ije/dyy121 (2018). 29955869 10.1093/ije/dyy121

[59] Lu, H., Gonsalves, G. S. & Westreich, D. Selection bias requires selection: The case of collider stratification bias. *Am. J. Epidemiol.* **193**(3), 407–409. 10.1093/aje/kwad213 (2023). 10.1093/aje/kwad213 PMC10911840 37939152

